# Patient Perceptions of Microbiome-Based Therapies as Novel Treatments for Mood Disorders: A Mixed Methods Study: Perceptions des patients sur les thérapies basées sur le microbiome pour les troubles de l’humeur : une étude à méthodes mixtes

**DOI:** 10.1177/07067437241234954

**Published:** 2024-02-28

**Authors:** Dina Moinul, Chenhui Hao, Gina Dimitropoulos, Valerie H. Taylor

**Affiliations:** 1 Department of Psychiatry, 70401 Cumming School of Medicine, 2129 University of Calgary, Calgary, AB, Canada; 2 Faculty of Social Work, 2129 University of Calgary, Calgary, AB, Canada

**Keywords:** depression, bipolar disorder, probiotics, prebiotics, fecal microbiota transplantation, microbiome-based therapies, dépression, trouble bipolaire, probiotiques, prébiotiques, transplantation de microbiote fécal, thérapies basées sur le microbiome

## Abstract

**Objective:**

Medications are critical for treating major depressive disorder (MDD) and bipolar disorder (BD). Unfortunately, 30% to 40% of individuals do not respond well to current pharmacotherapy. Given the compelling growing body of research on the gut-brain axis, this study aims to assess patient perspectives regarding microbiome-based therapies (MBT) such as probiotics, prebiotics, dietary changes, or fecal microbiota transplantation (FMT) in the management of MDD and BD.

**Methods:**

This single-centred observational study used quantitative and qualitative assessments to examine patient perceptions of MBT. Participants diagnosed with MDD or BD completed an anonymous questionnaire obtaining demographics, prior medication history, and symptom burden. Self-assessment questionnaires specific to each diagnosis were also used: Quick Inventory of Depressive Symptomatology Self-Report (QIDS-SR), Altman Self-Rating Mania Scale (ASRM), and General Anxiety Disorder Questionnaire (GAD-7). A logistic regression model analysed the association of MBT acceptance with disorder type, QIDS-SR, and GAD-7 scores. A bootstrap method assessed the proportion of MBT acceptance. The qualitative assessment consisted of 30-minute interviews to elicit perceptions and attitudes towards MBT.

**Results:**

The qualitative assessment achieved information power with *n* = 20. Results from the 63-item MBT questionnaire (*n* = 43) showed probiotics (37.2%) as the top choice, followed by FMT (32.6%), dietary change (25.6%), and prebiotics (4.6%). A majority of participants (72.1%) expressed willingness to try MBT for their mood disorder, however, logistic regression analysis did not identify statistically significant predictors for MBT acceptance among disorder type, QIDS-SR, and GAD-7.

**Conclusion:**

There is an increased focus on the gut microbiota's role in mood disorders’ etiology and treatment. Promising research and patient interest underscore the necessity for exploring and educating on patient perspectives and the factors influencing attitudes towards MBT.

## Résumé

**Objectif:**

Les médicaments sont essentiels pour traiter le trouble dépressif majeur (TDM) et le trouble bipolaire (TB). Malheureusement, de 30 à 40 % des personnes ne répondent pas bien à la pharmacothérapie actuelle. Compte tenu du nombre croissant de recherches sur l’axe intestin-cerveau (AI-C), la présente étude cherche à évaluer les perspectives des patients à l’égard des thérapies basées sur le microbiome (TBM) comme les probiotiques, les prébiotiques, les changements alimentaires, ou la transplantation de microbiote fécal (TMF) dans la prise en charge du TDM et du TB.

**Méthodes:**

La présente étude observationnelle monocentrique a utilisé des évaluations quantitatives et qualitatives afin d’examiner les perceptions des patients sur les TBM. Les participants ayant un diagnostic de TDM ou de TB ont rempli un questionnaire anonyme pour obtenir des données démographiques, des antécédents médicamenteux et la charge des symptômes. Des questionnaires d’auto-évaluation spécifiques à chaque diagnostic ont aussi été utilisés : Inventaire rapide de l'auto-évaluation des symptômes dépressifs (QIDS-SR), de l'échelle d'auto-évaluation de la manie d'Altman (ASRM) et du questionnaire sur les troubles anxieux généraux (GAD-7). Le modèle de régression logistique a analysé l'association de l'acceptation des TBM avec le type de trouble, les scores QIDS-SR et GAD-7.

**Résultats:**

L’évaluation qualitative a atteint le pouvoir d’information avec n = 20. Les résultats du questionnaire de TBM en 63 items (n = 43) ont révélé les probiotiques (37,2 %) comme premier choix, suivi de la TMF (32,6 %), du changement alimentaire (25,6 %), et des prébiotiques (4,6 %). Une majorité de participants (72,1 %) ont exprimé leur volonté d’essayer une TBM pour leur trouble de l’humeur, cependant, l’analyse de régression logistique n’a pas identifié de prédicteurs statistiquement significatifs de l’acceptation de la TBM chez le type de trouble, les QIDS-SR, et les GAD-7.

**Conclusion:**

Le rôle du microbiote intestinal dans l’étiologie et le traitement des troubles de l’humeur est d'un intérêt croissant. Les recherches fructueuses et l’intérêt des patients rendent nécessaire d’explorer et d’éduquer les perspectives des patients ainsi que les facteurs influençant les attitudes envers les TBM.

## Introduction

Behavioural-focused psychotherapies and/or pharmacotherapies such as antidepressants, mood stabilizers or antipsychotics are the most prescribed current treatments for major depressive disorder (MDD) or bipolar disorder (BD). However, 30% to 40% of patients do not respond to these strategies,^1^ while up to 55% experience side effects they rate as bothersome.^2^ There is, therefore, a need to explore new treatments for MDD and BD.

A compelling amount of research has shown that the intestinal microbiome, which refers to the communities of bacteria and fungi that reside in our gut, can also influence our brain, and subsequently, illnesses which have traditionally been identified as having a central etiology.^3^ The communication between the gut microbiome and the brain is now understood to be bidirectional and occurs through neural, inflammatory, and hormonal signalling pathways.^4^ The myriad of gut-brain axis (GBA) associations have sparked enthusiasm for exploring microbial manipulation as a potential therapeutic focus. This area represents a shift in understanding mood disorder etiology and introduces “psychobiotics” as a new class of treatment for mental illness.^5^

Growing evidence supports exploration of this area. Differences in the composition of the gut microbiome have been noted in many psychiatric illnesses and it is hoped that enhancing beneficial bacteria in the gut may be a therapeutic target.^6^ With respect to therapies, there is growing evidence supporting the potential of fecal microbial transplantation (FMT),^7,8^ while a meta-analysis of probiotic interventions on depressive symptoms showed statistically significant benefits.^9^ Several reviews have also indicated that dietary interventions can influence symptoms of mood disorders, although the exact types of diets and mechanism of change they cause have not been fully elucidated.^10,11^ Given the growing focus on the GBA, an ascertainment of the current level of knowledge around these therapies, and the way in which they are perceived by those with mental illness is important.

The primary purpose of our work, therefore, is to understand how individuals with a diagnosis of MDD or BD perceive the potential use of microbiome-based therapies (MBTs) as adjunctive in the management of mood disorders. The study objectives were to identify factors that may influence a participant's perception of MBT by examining: (i) which MBTs are preferred and what are the major facilitators and barriers in considering these MBTs as a treatment option? (ii) How do the type and severity of mood disorders or comorbid illnesses impact the perception of MBTs? (iii) To what extent does stigma with MBTs play into a patient's perception of these treatments?

## Methods

### Study Design

This study was conducted as a single-centred prospective observational study. It was composed of 2 parts: (1) a 30-minute online 63-item questionnaire and a series of self-report symptom rating scales and (2) a qualitative assessment through semi-structured recorded interviews of 20 to 30 minutes conducted on a subset of participants (*n* = 20) over Zoom.^12^ The interview was designed to gather insights into the participant's perceptions of MBT as a future potential therapeutic area. The questionnaire consisted of 3 parts, (i) demographic information, (ii) experiences with current diagnoses, and (iii) perception of MBT, and was presented to participants via online survey software Qualtrics.^13^ This work received ethical approval from the Research Ethics Board at the University of Calgary (REB21-0443).

### Recruitment

Participants were recruited via an established patient consent to contact data registry and were recruited from June 11, 2021, to April 26, 2022. The study's inclusion criteria were for adults ≥18 years of age with a diagnosis of MDD or BD, who had access to a phone, computer, and stable internet connection, and who read and spoke English. Exclusion criteria included participants who had active suicidal ideation or were experiencing psychosis or mania.

### Data Collection

Zoom^12^ was utilized to conduct qualitative interviews using a semi-structured interview guide developed in collaboration with the team. The interview guide examined patients’ experience with mental illness, current and previous treatments, and knowledge, understanding and willingness to try MBTs such as probiotics, prebiotics, dietary change, and FMT. Recruitment was ongoing until no new information (information power^14^) was obtained (*n* = 20). The interviews were digitally audio-recorded, transcribed verbatim using Zoom.com, and anonymized and verified by the project team.

The 63-item MBT questionnaire and self-report symptom rating scales were collected from participants (*n* = 43) from June 2021 to April 2022. These evaluations asked participants to retrospectively rate their perception of MBT and current symptoms. Results from standardized self-assessment questionnaires were also obtained.

### Interventions

The perceptions of the MBT questionnaire administered were created using a questionnaire developed and validated to assess attitudes towards FMT in participants with ulcerative colitis, modified and used with permission from Kahn et al.^15^ The survey included 38 questions about FMT, disease activity, clinical effectiveness, and satisfaction with current treatments. For our study, we included an additional 30 questions. These questions explored participant willingness and experiences with 4 MBTs and asked about comorbid mental and physical illnesses and current psychotropic medication use. The survey was validated and refined with healthy adult volunteers to gauge readability, clarity, and literacy level. Participants were also asked to complete validated self-assessment questionnaires to detail current symptom burden: the Quick Inventory of Depressive Symptomatology Self-Report (QIDS-SR),^16^ the Altman Self-Rating Mania Scale (ASRM),^17^ and the General Anxiety Disorder Questionnaire (GAD-7).^18^

### Data Analysis

A thematic analysis approach was employed to identify patterns and themes within the data, allowing insights into the main categories that characterize the phenomenon being investigated.^19^ The transcriptions were inductively analysed through an interactive process after being imported to a qualitative data analysis programme, NVivo 12.^20^ The initial stage of data analysis involved identifying key concepts and using small, descriptive codes. These codes were then integrated to generate comprehensive themes. These emergent themes were further scrutinized within and between groups (cohorts) to obtain an in-depth comprehension of the experiences and ensure the credibility, validity, and confirmability of the data.^21^ Additionally, memos were maintained to record any significant analytical modifications and personal thoughts, thereby ensuring confirmability and dependability of the study.^22^

### Statistical Analysis

Our study aimed to investigate the impact of the type and severity of mood disorders on MBT perceptions. We hypothesized that neither the disorder type nor the severity of the symptoms would significantly influence MBT acceptance. To test this hypothesis, we employed a logistic regression model.^23^ Responses were initially categorized into “Yes” (*n* = 31), “No” (*n* = 1), and “Unsure” (*n* = 11). Given the limited number of responses in the “No” category, it was integrated with “Unsure” to improve the stability and reliability of the regression analysis. The independent variables were disorder type, QIDS-SR, and GAD-7 score, but due to data incompleteness, we excluded ASRM from the final analysis, resulting in a total sample size of *n* = 43 for the regression analysis. Prior to fitting the model, we performed diagnostic tests to verify the logistic regression assumptions. Multicollinearity among the independent variables was evaluated using the variance inflation factor method, and the goodness of fit for the model was assessed by the area under the curve (AUC). Additionally, a Likelihood ratio test was conducted to compare the full model against a null model without the predictors to determine if the inclusion of disorder type, QIDS-SR, and GAD-7 scores significantly improved the model's fit. To further validate the observed acceptance rate favouring MBT, we used a bootstrap method^24^ to re-estimate the participant's acceptance proportion. This resampling with the replacement method allowed us to create an empirical distribution of the acceptance proportion without parametric assumption. All statistical analysis was performed in R statistical software (version 4.3.1).^25^

## Results

### Qualitative Evaluation

The duration of the qualitative interviews (*n* = 20; *F* = 16 [80%]) ranged from 21:31 to 73:14 minutes (mean: 34:57 minutes). Regarding diagnosis, 35% had MDD and 65% BD, as shown in Table 1.

**Table 1.** Patient Demographics and Mental Health Disorder Diagnosis for Quantitative (*n* = 43) and Qualitative Participants (*n* = 20).

Gender	Major depressive disorder	Bipolar disorder
Quantitative study participants		
Female	16 (37.2%)	16 (37.2%)
Male	4 (9.3%)	6 (14.0%)
Other	0 (0.0%)	1 (2.3%)
Total	20 (46.5%)	23 (53.5%)
Qualitative study participants		
Female	7 (35.0%)	9 (45.0%)
Male	0 (0.0%)	4 (20.0%)
Other	0 (0.0%)	0 (0.0%)
Total	7 (35.0%)	13 (65.0%)

Several themes and subthemes emerged from the data analysis. These themes are presented in Table 2 and in further detail below. Quotes presented here have been anonymized and edited only for grammar and readability.

Theme 1: Lack of Resources and Knowledge on MBT
  Theme 1.1: Limited availability of essential resources

**Table 2.** Qualitative Data Illustrating Themes, Sub-Themes and Illustrative Participant Quotes Extracted From Interviews.

Theme	Subtheme	Illustrative quote
Theme 1: Lack of resources and knowledge on MBT		
	Limited availability of essential resources	Living with bipolar disorder is a constant struggle, but I've heard whispers about the potential of microbiome-based therapies […] It leaves us feeling lost and without the essential knowledge we need to improve our mental health. (Participant 11)
	Inadequate familiarity with MBT and its potential advantages	The limited awareness of these other treatments hinders our ability to make informed decisions about our ever-so-changing health. (Participant 12)
Theme 2: Emotional impact, concerns and uncertainty surrounding MBT		
		[…] triggers a range of emotions within me, including anxiety. I worry about the potential risks, the impact on my overall health, and whether it will truly help alleviate my condition […] I’ve just jumped from medication to medication which have skipped my symptoms and made it worse, so it is traumatic for me to try new medication. (Participant 4)
Theme 3: Practical challenges of MBT		
	Financial ramifications and anxieties pertaining to the exorbitant cost of MBT	The cost of [MBT] often leads to major financial stress. I mean the potential for improved health comes with a hefty price tag, leaving us to grapple with ‘how do we go ahead and pursue these treatments? (Participant 13)
Theme 4: Practical challenges of current psychotherapy medications	Disruption to daily routines, livelihoods, and established schedules	I've been thinking about maybe tweaking my diet. Anything to help me. It's like, these mood swings and daily chaos are draining. (Participant 11)

*Note*: MBT = microbiome-based therapies.

There is a need for comprehensive and accessible information that explains the concept of MBT, its underlying mechanisms, and its potential advantages for mental health conditions. This information should be readily available through various mediums, including online platforms, educational materials, and support networks, allowing patients to learn about MBT and make informed decisions about their treatment options.

Living with depression, I'm constantly searching for new approaches to manage my symptoms. The limited availability of essential resources for microbiome-based therapies feels like a roadblock, preventing us from exploring potential treatments that could offer hope and a renewed sense of well-being. (Participant 9)

Theme 1.2: Inadequate familiarity with MBT

Insufficient familiarity with MBT hinders patients from exploring the MBT space. Participants in this study expressed the need to address this issue which requires comprehensive education and dissemination of accurate information to patients, health-care providers, and the public. Empowering health-care professionals with robust knowledge of MBT and its scientific evidence is essential. Fostering peer support and advocacy groups to share experiences and resources would enhance patient's confidence in MBT. Furthermore, investing in targeted research and well-designed clinical trials specific to MBT for BD and MDD will provide critical evidence to determine the appropriateness of MBTs for some individuals.

I wish more doctors and healthcare professionals were familiar with the potential advantages of these treatments. It's disheartening to encounter skepticism or lack of awareness from others, which prevents from accessing innovative approaches to treatment. (Participant 9)

Many participants also expressed a desire to deepen their knowledge about MBT as there was minimal understanding of the connections it bears with the GBA.

To be honest, I'd like to hear more from you about the fecal transplantation, because I don't honestly know how that works and sort of what's involved, what's the procedure, and you know, how do you evaluate effectiveness and so on. (Participant 14)

Theme 2: Emotional Impact, Concerns and Uncertainty surrounding MBT

Participants expressed a complex array of emotions and concerns regarding MBT, encompassing both trepidation and heightened uncertainty. Feelings of distress and anxiety, coupled with the pervasive stigma linked to alternative treatments, may serve as formidable barriers, dissuading patients with MDD and BD from embracing MBT.

I wish there was less stigma surrounding MBT treatments […] I talk to my family about it just to feel judged or invalidated for considering innovative approaches that could potentially improve my health and well-being. (Participant 14)

Some participants in the study who were exploring treatments for MDD and BD expressed apprehension about MBT. Anticipated side effects and uncertainties heighten anxieties, despite not yet experiencing MBT. Previous treatment side effects, occasionally difficult, contribute to overall hesitancy towards unconventional therapies.

Choosing [MBT] for my mood [disorder] is tricky. Switching from my current meds, I stress about potential rough patches, even if the new approach works. The fear of any side effects with new treatments adds to the hesitation. It's a tough call. (Participant 3)

Participants faced a significant challenge in identifying suitable therapies for their mood disorders, with a shared inclination towards natural alternatives such as dietary changes over conventional medications. This preference for more holistic approaches to the whole body was consistently expressed, highlighting a collective willingness to explore alternatives such as MBT, provided they are proven safe and effective.

If I could find something a little bit better that isn't medication, whether it be food change or something else, I would try it. (Participant 7)

If [the MBT] are beneficial and they're proven safe and effective I don't have a problem trying things out that could eliminate a pill to make it more natural from what I eat, as opposed to putting it into my system. (Participant 15)

Theme 3: Economic Impact of MBT on Patients

The costs of MBT impose significant financial burdens and anxieties on patients. Access to these treatments becomes challenging, fueling frustration and helplessness. The added financial strain amplifies the already overwhelming stress of managing their conditions, jobs, and lifestyle jeopardizing their mental well-being. These circumstances force patients to make agonizing choices between MBT and essential financial obligations.

The cost [of probiotics] would be the biggest factor for me, definitely! It can feel overwhelming. The fear of accumulating medical expenses and the strain it puts on finances adds a layer of stress. So, I wouldn’t be able to continue the probiotics because I couldn’t afford it. (Participant 12)

Theme 4: Balancing MBT and Autonomy in Daily Well-being

There was enthusiasm for MBTs, especially when other treatments were inadequate. Exploring alternative approaches such as MBT was hoped to offer relief by addressing these challenges and potentially mitigating the exacerbation of stress and disruptions in daily life.

You know, living with this [mood disorder] it messes up everything—work, relationships, just daily life. It's this ongoing struggle with mood swings, fatigue, and brain fog. But hey, thinking about trying something new, like a different diet, feels like grabbing a bit of control back. It's not a cure-all, but it's a little something to maybe make life's craziness a bit more manageable. (Participant 17)

### Quantitative Results

We collected patient perspectives (*n* = 43; *F* = 32 [74.4%]) on their willingness to try MBT and to rank their MBT preference. In this sample population, 46.5% had MDD and 53.5 had BD regarding diagnosis, as shown in Table 1. The majority of participants (31 [72.1%]) are willing to try MBT with 17 MDD patients (39.5%) and 14 BD patients (32.6%) while 11 patients (25.6%) are unsure about MBT with 3 MDD patients (7.0%) and 8 BD patients (18.6%), as detailed in Table 3. The resulting rankings are quantified in Table 4, which provides a comparison of the 4 therapies highlighted in this paper for patients diagnosed with MDD and BD separately. The therapies are ranked from 1 (most-preferred) to 4 (least-preferred). Among the 4 treatments, 40% of patients with MDD preferred FMT. For patients with BD, 47.8% showed the most interest in Probiotics. However, FMT was the least preferred for most patients with either diagnosis.

**Table 3.** Patient Self-Reported Willingness to Try Microbiome-Based Therapies (MBT) Based on Mental Health Disorder (*n* = 43).

Willingness to try MBT	Major depressive disorder	Bipolar disorder	Total
Yes	17 (39.5%)	14 (32.6%)	31 (72.1%)
No	0 (0.0%)	1 (2.3%)	1 (2.2%)
Unsure	3 (7.0%)	8 (18.6%)	11 (25.6%)

**Table 4.** Patient Ranking of Preferred MBT (Most-1 to Least-4) Based on Each Mood Disorder (*n* = 43).

MBT	Major depressive disorder (*n* = 20)				Bipolar disorder (*n* = 23)			
Ranking	1	2	3	4	1	2	3	4
Probiotics	5 (25.0%)	5 (25.0%)	5 (25.0%)	5 (25.0%)	11 (47.8%)	6 (26.1%)	3 (13.0%)	3 (13.0%)
Prebiotics	2 (10.0%)	7 (35.0%)	11 (55.0%)	0 (0.0%)	0 (0.0%)	11 (47.8%)	10 (43.5%)	2 (8.7%)
Dietary change	5 (25.0%)	7 (35.0%)	4 (20.0%)	4 (20.0%)	6 (26.1%)	4 (17.4%)	8 (34.8%)	5 (21.7%)
FMT	8 (40.0%)	1 (5.0%)	0 (0.0%)	11 (55.0%)	6 (26.1%)	2 (8.7%)	2 (8.7%)	13 (56.5%)

*Note*. MBT = microbiome-based therapies; FMT = fecal microbiota transplantation.

Percentages do not add up to 1 due to rounding.

**Figure 1.**
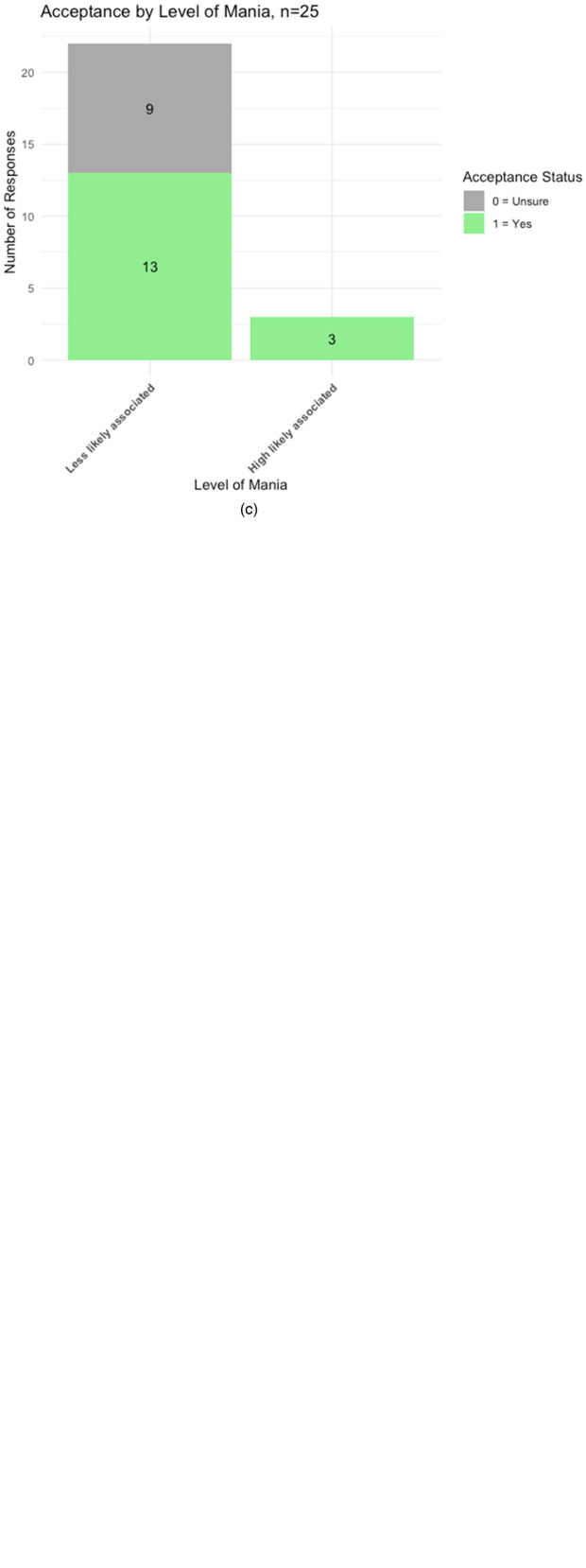
Patient response distribution by categories of 3 clinical measures. (a) Response frequency by categories of QIDS-SR, *n* = 43. (b) Response frequency by categories of GAD-7 scale, *n* = 43. (c) Response frequency by categories of ASRM, *n* = 25.

*Note*: QIDS-SR = Quick Inventory of Depressive Symptomatology Self-Report; GAD-7 = General Anxiety Disorder Questionnaire; ASRM = Altman Self-Rating Mania Scale.

Figure 1 presents bar plots visualizing the distribution of patient responses to MBT, stratified by 3 clinical measures: QIDS-SR (Figure 1(a)), GAD-7 (Figure 1(b)) and ASRM (Figure 1(c)). The “Yes” responses are distributed at all depression severity levels and mainly at lower levels for Anxiety and Mania, while the “Unsure” votes are mainly distributed at lower symptom severity levels for all 3 measures.

Using a logistic regression model, we assessed the effects of disorder type, QIDS-SR, and GAD-7 scores on the likelihood of participants accepting MBT. The model results did not yield any statistically significant predictors for the MBT acceptance among the 43 participants. This aligned with our hypothesis that patient opinions of MBT are not influenced significantly by the 2 disorder types and symptom severity. Additionally, a Likelihood ratio test showed an improvement in the full model (χ²(3) = 6.714, *p* = 0.082) compared to the null model, but this was not significant. Despite the lack of significant predictors, the goodness of fit for the model as assessed by AUC was 0.720, indicating a moderate predictive ability overall.

Further analysis using a bootstrap method with 2000 resamples yielded an estimated mean acceptance proportion of 0.722, which closely matched the observed sample proportion of acceptance (0.721). The 95% confidence interval of the mean acceptance proportion [0.58 to 0.86] excluded the no preference value of 0.5. This strengthens the evidence that the true acceptance rate is above the threshold of random chance, indicating a preference in favour of MBT.

## Discussion

### Which Factors Affect an Individual's Decision to Try MBT?

Despite no statistically significant predictors found in our quantitative analysis, the rankings derived from patient perspectives revealed a clear inclination towards MBT across various symptom severities. The bootstrap method demonstrated a significant deviation from neutrality, indicating a substantial preference for MBT among participants. This collective inclination towards MBT suggests a promising inherent appeal to exploring MBT as a potential therapy in the future. As this field moves forward, however, there are challenges that will need to be addressed to improve acceptability.

In our study, cost emerged as a barrier to MBT, with some participants expressing hesitancy to the idea due to financial concerns. A persistently high level of food insecurity is common among individuals with mental illness,^26,27^ and while we do not know precisely which types of diets are the most beneficial in improving the gut microbiome, the most common candidates involve a Mediterranean diet or 1 high in fibre, or the presence of fermented superfoods. These types of diets can be very expensive. Long-term use of probiotics can also be costly and are not reimbursed by health care coverage plans, and the cost factor has been identified as an issue to consider when recommending these types of treatments, especially given the lack of well-designed trials in this area.^28^ However, dispelling stereotypes about healthy diets is crucial. Foods rich in fibre, including onions, garlic, leeks, bananas, beans, peas, and lentils, are both nutritious and affordable. Healthy eating does not mandate costly interventions such as over-the-counter probiotics or private FMT. Known for its capacity to alter gut microflora, FMT shows better evidence for populations with symptoms, while dietary modification and probiotics may be more suitable for maintaining wellness.^29^ To address these concerns, education plays a paramount role. There is a need to provide education on implementing cost-effective strategies for healthy eating, highlighting evidence for various MBTs.

Most of the ongoing work in this field pertains to work required in education and knowledge translation. While a growing body of literature suggests a connection between gut microbiota and mood disorders, some participants were still unsure about the link between these 2 seemingly disparate systems and how MBTs could impact symptoms of brain-based illnesses. To address this knowledge gap effectively, it is crucial to integrate robust research findings with education. This promotes a nuanced understanding of the intricate connection between GBA and MBTs for practitioners and patients.^30^

Side effects and safety concerns were also cited as potential factors that could impact an individual's decision to try MBT, especially as gastrointestinal issues are part of the side effects of many current pharmacotherapies.^2^ While probiotics and dietary modifications are generally considered safe, there are still concerns about the safety and efficacy of FMT and longitudinal work is needed on all MBTs to address these concerns and ensure the safety and efficacy of MBT.

Stigma is a well-documented obstacle to health-seeking behaviour, participation in care, and adherence to treatment across a wide spectrum of health disorders globally. Individuals with a mental illness may feel embarrassed, ashamed, or face concerns over public perception when considering nontraditional or novel treatments such as MBTs, thereby creating a significant barrier to exploring these areas.^31^ The concept of microbial manipulation, such as FMT, may be too unusual for some, and the way it is altered may be off-putting.

### Limitations

A limitation is the study's single-centre design, which limits the generalizability of the findings to other populations, especially given that sampling occurred from an academic centre where participants are potentially more likely to be exposed to alternative or research treatments. Further research with larger samples and multiple centres is necessary to determine the broader acceptance and feasibility of MBT as a treatment option for MDD and BD. It is important to also ensure a wide range of sex, gender, and ethnicity is represented in the sampling. Sampling not just those looking to take a treatment, but prescribers as well, would also be important to understand practitioners' views on MBT. While this study focused on patients with MDD and BD, future studies could research the perceptions of other patient populations.

Additionally, participants self-selected for the interview process, which may reflect a predisposition to openness, potentially correlating with an increased acceptance of novel treatments. The qualitative assessment portion of the study also relied on self-reported attitudes and perceptions, which may be subject to recall and social desirability biases.

## Conclusion

Research into the gut microbiota's impact on mental health is a burgeoning field. Although many questions remain unanswered, researchers are gaining access to a wealth of data describing the structure and function of the gut microbiome. Issues such as lack of education and miseducation persist in this evolving field, underscoring the importance of engaging with patients and practitioners to develop a more effective approach to knowledge translation. While further studies are necessary to fully understand the benefits of using MBT interventions, it is also important to understand how to engage with those with lived experience regarding a therapeutic modality that to some may be very novel.

## Supplemental Material

sj-docx-1-cpa-10.1177_07067437241234954 - Supplemental material for Patient Perceptions of Microbiome-Based Therapies as Novel Treatments for Mood Disorders: A Mixed Methods Study Supplemental material, sj-docx-1-cpa-10.1177_07067437241234954 for Patient Perceptions of Microbiome-Based Therapies as Novel Treatments for Mood Disorders: A Mixed Methods Study by Dina Moinul, Chenhui Hao, Gina Dimitropoulos and Valerie H. Taylor in The Canadian Journal of Psychiatry

sj-docx-2-cpa-10.1177_07067437241234954 - Supplemental material for Patient Perceptions of Microbiome-Based Therapies as Novel Treatments for Mood Disorders: A Mixed Methods Study Supplemental material, sj-docx-2-cpa-10.1177_07067437241234954 for Patient Perceptions of Microbiome-Based Therapies as Novel Treatments for Mood Disorders: A Mixed Methods Study by Dina Moinul, Chenhui Hao, Gina Dimitropoulos and Valerie H. Taylor in The Canadian Journal of Psychiatry
