# Supplementary material for: Patient Perceptions of Microbiome-Based Therapies as Novel Treatments for Mood Disorders: A Mixed Methods Study: Perceptions des patients sur les thérapies basées sur le microbiome pour les troubles de l’humeur : une étude à méthodes mixtes
Source: Can J Psychiatry. 2024 Feb 28;69(7):503–12. doi: 10.1177/07067437241234954 (PMC11168347; doi:10.1177/07067437241234954)
Supplement: sj-docx-1-cpa-10.1177_07067437241234954 - Supplemental material for Patient Perceptions of Microbiome-Based Therapies as Novel Treatments for Mood Disorders: A Mixed Methods Study [file sj-docx-1-cpa-10.1177_07067437241234954.docx]

**Supplementary Material**

**Table S1.** Patient diagnosis of MDD and BD self-reported severity based on QIDS-SR_16_, ASRM, and GAD-7 (n=43).

| **QIDS-SR_16_** | | **ASRM** | | **GAD-7** | |
| --- | --- | --- | --- | --- | --- |
| Number of Patients (n=43) | Severity | Number of Patients (n=43) | Severity | Number of Patients (n=23) | Severity |
| 8 (18.6%) | No Depression | 20 (46.5%) | Minimal Anxiety | 21 (87.5%) | Less likely to be associated with significant symptoms of mania |
| 15 (34.9%) | Mild Depression | 9 (20.9%) | Mild Anxiety | 3 (12.5%) | High probability of a manic or hypomanic condition |
| 6 (14.0%) | Moderate Depression | 6 (14.0%) | Moderate Anxiety |  |  |
| 9 (20.9%) | Severe Depression | 8 (18.6%) | Severe Anxiety |  |  |
| 5 (11.6%) | Very Severe Depression |  |  |  |  |

**Table S2.** Logistic regression model output.

| **Variable** | **Estimate** | **Std. Error** | **Z value** | **Pr(>\|z\|)** |
| --- | --- | --- | --- | --- |
| **(Intercept)** | 2.4155 | 1.5580 | 1.550 | 0.1211 |
| **Disorder type** | -0.9655 | 0.8014 | -1.205 | 0.2283 |
| **QIDS-SR16** | -0.1052 | 0.1014 | -1.037 | 0.2997 |
| **GAD-7** | 0.1937 | 0.1158 | 1.673 | 0.0944 |
| Signif. codes: 0 ‘***’ 0.001 ‘**’ 0.01 ‘*’ 0.05 ‘.’ 0.1 ‘ ’ 1  Null deviance: 50.918 on 42 degrees of freedom  Residual deviance: 44.204 on 39 degrees of freedom  AIC: 52.204 | | | | |
