# Supplementary material for: Patient Perceptions of Microbiome-Based Therapies as Novel Treatments for Mood Disorders: A Mixed Methods Study: Perceptions des patients sur les thérapies basées sur le microbiome pour les troubles de l’humeur : une étude à méthodes mixtes
Source: Can J Psychiatry. 2024 Feb 28;69(7):503–12. doi: 10.1177/07067437241234954 (PMC11168347; doi:10.1177/07067437241234954)
Supplement: sj-docx-2-cpa-10.1177_07067437241234954 - Supplemental material for Patient Perceptions of Microbiome-Based Therapies as Novel Treatments for Mood Disorders: A Mixed Methods Study [file sj-docx-2-cpa-10.1177_07067437241234954.docx]

**Supplementary Material**

**Semi Structured Interview Guide**

**Introduction**

“Thank you for participating in this interview. We are interviewing you to gain a better understanding of patients’ thoughts on new microbiome-based therapies for depression or bipolar disorder. We are interested in hearing about your experiences with your mental illness and current treatments as well as your views and knowledge around microbiome-based therapies.

This interview should take no more than thirty minutes depending on how much you would like to share. With your permission, I would like to record the audio of this interview, so I don’t miss anything. If you would like, we can turn the camera on as well, but your video will also be recorded then. All responses and audio/video recordings will be kept confidential, only used by research team members and keep you unidentified by assigning you a number code. You may decide to decline answering or stop the interview at any point for any reason. Do you have any questions?

May I turn the recorder on?

1. **Topic: Previous Mental Illness Experience**

**Guiding Question: Can you tell me about your experiences with your mental illness?**

- **Prompts:** What diagnoses have you had to deal with? What has been challenging about this diagnosis?
- **Prompts:** How are these symptoms affecting your day-to-day life? (School, work, home life etc) What are the most or least concerning symptoms?

**Guiding Question: What treatments have you pursued in the past or are currently trying out?**

- **Prompts:** Have you tried talk therapy, medication, other forms of psychiatric treatment, or alternative medicine like yoga, vitamins, or acupuncture? What are the advantages and disadvantages of any of these treatments? What are your concerns when trying these different treatments i.e., safety, efficacy, time, money etc.?

**Guiding Question: What are your thoughts about current treatments for your mental illness?**

- **Prompts:** Are you content with the treatments available to you?
- **Prompts:** What are your thoughts on exploring alternative forms of treatment that are new to you? How comfortable are you with trying less conventional therapies? Is there anything you’re immediately apprehensive about?

1. **Topic: Patient Knowledge and Understanding of Microbiome Based Therapies**

In the first part of this study, you were asked to complete a survey on your thoughts around microbiome-based therapies. The following questions will ask similar questions. As a reminder, these treatments include: (1) probiotics, healthy live bacteria introduced to the body for a health benefit, (2) prebiotics is a non-digestible fiber that helps promote growth of healthy bacteria in the body, (3) dietary changes towards increasing fiber and following a plant-rich Mediterranean-like diet to promote growth of healthier gut bacteria and reduce stress levels and (4) FMT, the transfer bacteria from a healthy donor to a person with a disease such as depression or bipolar disorder.

**Guiding Question: As mentioned in the questionnaire portion of this study, new microbiome-based therapies for depression and bipolar disorder are being investigated. Have you any prior knowledge or experience around probiotics, prebiotics, dietary changes or fecal microbiota transplantation?**

- **Prompts:** Have you heard of any of these therapies before, and if so, which ones? What do you know about these therapies? Where did you learn about these treatments from? (e.g., Physician, friends/family, social media, online)

**Guiding Question: How do you feel about microbiome-based therapies being used as new treatments for mental illnesses?**

- **Prompts:** What is your current understanding of these treatments? Is there anything you find alluring about these treatments? Is there anything you think you would not like?
- **Prompts:** Do you have any thoughts on using these therapies over other pharmacological treatments? Do you think one may be more beneficial over another? Why?

1. **Topic: Patient Willingness to Try Microbiome Based Therapies**

**Guiding Question: How willing would you be to try any of these therapies?**

- **Prompts:** Is there any treatment you would be willing to try over another? Tell me more about what appeals to you most about this treatment.
- **Prompts:** Is there any you would not be willing to try at all? Why?

**Guiding Question: Are there any major overarching concerns you would have with trying any of these therapies?**

- **Prompts:** For example, how would you feel about incorporating any of these treatments into your life? How realistic is it for you to take additional pills of probiotics, prebiotics or fecal microbiota transplantation along with your regular medication? How realistic is a dietary lifestyle change for you?
- **Prompts:** Do you have any concerns about safety, efficacy, time, money etc.? To what extent do these concerns impact your decision in trying a microbiome-based therapy?

1. **Topic Patient Fear of Stigma Towards Microbiome Based Therapies**

**Guiding Question: How would you feel about openly trying a new mental illness treatment like a microbiome-based therapy?**

- **Prompts:** How would you feel about telling your friends, family or coworkers that you are trying new treatment? How comfortable would you feel telling them you are trying microbiome-based treatment, with specifics on which one? Is there one treatment you’d be more or less comfortable sharing about? If so, why?
- **Prompts:** How would the opinion of others around you effect your desire to try one of these new treatments? What factors would you be most reluctant sharing about?

1. **Conclusion**

**Guiding Question: With your current understanding of microbiome-based treatments, would you recommend these treatments to another patient with a similar diagnosis?**

- **Prompts:** Why or why not would you recommend this?

**Guiding Question: Is there anything else you would like to add about how your thoughts on microbiome-based therapies that we haven’t already spoken about?**

Thank you very much for your time and sharing your thoughts with us.
